# Effect of two deproteinized bone graft materials for socket preservation: a clinical and histological study

**DOI:** 10.3389/froh.2025.1630504

**Published:** 2025-07-09

**Authors:** Marwa Madi, Nasser S. Al-Naief, Adel S. Alagl

**Affiliations:** ^1^ Department of Preventive Dental Sciences, College of Dentistry, Imam Abdulrahman Bin Faisal University, Dammam, Saudi Arabia; ^2^ Department of Biomedical Dental Sciences, College of Dentistry, Imam Abdulrahman Bin Faisal University, Dammam, Saudi Arabia

**Keywords:** Bio-Oss, NuOss, deproteinized bone graft, socket preservation, CBCT, histological findings

## Abstract

**Objective:**

To evaluate dimensional changes and new bone formation using two deproteinized bovine bone minerals, NuOss and Bio-Oss, in socket preservation.

**Materials and methods:**

Eighteen patients (6 males, 12 females; aged 23–45 years) requiring posterior tooth extraction were enrolled. Eighteen extraction sockets were augmented with either NuOss or Bio-Oss and covered with a collagen membrane. After six months, Cone Beam Cephalometry (CBCT) assessed dimensional changes in buccolingual width and buccal bone thickness. Bone core biopsies were obtained during implant placement and decalcified for histomorphologic examination. Statistical analysis compared dimensional changes and histomorphometric parameters between groups.

**Results:**

All experimental sites healed uneventfully, with complete soft tissue healing within four weeks and successful implant placement. CBCT scans showed comparable, non-significant dimensional reductions. Histomorphologic examination revealed lamellar cortical bone and osteoid trabeculae with partial to optimal integration. NuOss demonstrated significantly higher new bone formation (52.5 ± 2.5%) compared to Bio-Oss (37.5 ± 2.5%; *p* = 0.0021), with lower residual graft material (27.5 ± 2.5% vs. 42.5 ± 2.5%; *p* = 0.0018). Bio-Oss grafted cases exhibited more pronounced inflammatory cell infiltration. Soft tissue proportions were similar between groups (NuOss: 22.5 ± 2.5%, Bio-Oss: 17.5 ± 2.5%; *p* = 0.0892).

**Conclusion:**

Both NuOss and Bio-Oss showed positive bone regeneration effects. However, NuOss demonstrated more favorable biocompatibility, with less inflammation and improved bone integration than Bio-Oss.

## Introduction

The alveolar process is a tooth-dependent structure; hence, the teeth removal results in a marked reduction in its dimension due to the modeling process (1). Thus, both the horizontal and the vertical dimensions will be markedly diminished. At such sites, the buccal wall of the post-extraction socket will become more reduced than the corresponding lingual/palatal bone wall (2).

It was suggested by Paolantonio et al. (3) that implants placed in the fresh extraction socket may counteract post-extraction ridge alterations. Findings from animal experiments (4, 5) and studies in humans revealed, however, that so-called “immediate implants” failed to offset resorption of the buccal and palatal/lingual bone walls (6, 7). Subsequently, alveolar ridge preservation technique (ARP) has been proposed, in which bone substitutes are placed in the extraction socket following tooth removal (8). Although evidence shows that this technique has its benefit in limiting the resorption process, it does not overcome this problem (9–11).

The selection of an optimal biomaterial for ridge preservation remains a subject of ongoing debate in dental research. While bone graft materials serve primarily through their osteoconductive properties to maintain space and promote bone growth (12, 13), their comparative efficacy varies. Bone substitutes in dentistry serve the primary purpose of regenerating or filling defective bone areas. Xenografts, which demonstrate low resorption rates and osteoconductive properties, represent one category of these substitutes (14). These materials, derived from bovine or porcine sources, have gained popularity in ridge preservation procedures due to their accessibility and ease of handling (15). Among xenografts, demineralized bovine bone has seen wider adoption compared to porcine-derived materials (16). Bio-Oss, one of the most extensively studied xenografts, undergoes processing to replicate natural bone structure while removing organic components (16, 17). Human histological studies have demonstrated that while xenograft placement may extend the healing period, it effectively maintains socket and ridge dimensions compared to non-grafted sites (18).

Deproteinized bovine bone has shown promise in alveolar reconstruction (19, 20), though some studies have reported limitations including incomplete preservation and excessive connective tissue proliferation (21, 22). Alternative materials such as hydroxyapatite and calcium sulphate have demonstrated success in preservation procedures (23). However, autogenous bone continues to be considered the gold standard, showing superior outcomes compared to allograft materials (24). Nevins et al. (25) concluded in his clinical study that fresh extraction sockets in the maxillary front tooth region that were grafted with a deproteinized bovine bone mineral demonstrated less loss of the buccal plate of the ridge than non-grafted control sites. Measurements performed in histological sections demonstrated that socket grafting with the use of deproteinized bovine bone mineral made it possible to preserve most of the dimension of ridge.

In a systematic review on ridge preservation after tooth extraction, Vignoletti et al. (8) concluded that socket grafting with biomaterial may result in less vertical and horizontal contraction of the bone crest, but also that there is no clear guideline supported by scientiﬁc evidence to indicate the type of biomaterial to be used. Avila-Ortiz et al. (26) The application of particulate xenogenic or allogenic materials covered with an absorbable collagen membrane or rapidly absorbable collagen sponge seems to be associated with the most favorable outcomes in terms of horizontal ridge preservation. The aim of this clinical study was to evaluate the dimensional changes and new bone formation of two deproteinized bovine bone minerals, Bio-Oss and NuOss, used for socket preservation.

## Material and methods

This observational study was conducted at the College of Dentistry, Imam Abdulrahman Bin Faisal University (IAU), to evaluate outcomes following socket preservation procedures using deproteinized bone graft materials. The study protocol received ethical approval from the institutional review board (IRB-2025-02-0125) and adhered to the principles of the Declaration of Helsinki (2013) (27). All participants provided informed consent after a detailed explanation of the study procedures.

### Patient selection

Patients who seek socket preservation procedures within the past six months at the College of Dentistry, were identified and recruited for the study. Inclusion criteria comprised systemically healthy, non-smoking patients aged 21–50 years, with no bone metabolism-affecting medications and good oral hygiene (full-mouth plaque score <20%). Patients were excluded if they had stage III/IV periodontitis or active periodontal lesions, acute infection, or severely resorbed sockets (buccal bone heights <5 mm), smokers (>10 cigarettes/day), or current pregnancy.

Indications for tooth extraction included caries, failed endodontic treatment, unrestorable teeth, and tooth fracture. All patients underwent comprehensive periodontal examination and initial periodontal therapy, including oral hygiene instructions, scaling, and root planing, at least 4 weeks prior to the surgical procedure. Pre-operative CBCT scans (KODAK 9500 Cone-Beam 3D System, Carestream, Rochester, NY, USA) from patient records were analyzed to assess pre-extraction tooth conditions. Existing clinical records included inter-maxillary relationship impressions that had been used to fabricate reference stents for measuring horizontal bone dimensional changes post-extraction. Sample size estimation was based on the mean dimensional changes of the socket following 6 months of healing, with a standard deviation of 10%, alpha = 0.05, and power = 80%. A minimum of 9 sockets per group (18 total) was determined as adequate.

### Surgical protocol

Pre-existing CBCT scans (KODAK 9500 Cone-Beam 3D System) from patient records were analyzed to measure: Buccal wall thickness at 2 mm from the crest, and Buccolingual width at the extraction site. All patients underwent standardized socket preservation procedures following tooth extraction. Patients were allocated to either the Bio-Oss or NuOss group according to routine clinical protocols followed by the treating clinicians. However, the evaluators were blinded to the groups during the analysis. Under local anesthesia (2% lidocaine with 1:100,000 epinephrine), a sulcular incision was conducted using a 15c surgical blade. Atraumatic tooth extraction was then performed using a combination of periotome, tooth separation, and dental forceps with careful attention to preserve the integrity of the buccal plate. Following extraction, sockets were thoroughly debrided using curettes to remove any residual soft tissue or granulation material.

**Figure 1:**
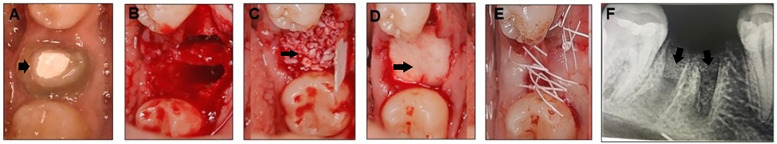
Mandibular right first molar indicated for extracted (black arrow) **(A)**, extraction socket **(B)** socket augmented with nuOss graft materials (black arrow) **(C)** and covered with a conform collagen membrane (black arrow) **(D)** sutured with 4-0 PTFE **(E)**, and post-operative periapical radiograph showing the grafted socket (black arrow) **(F)**.

**Figure 2:**
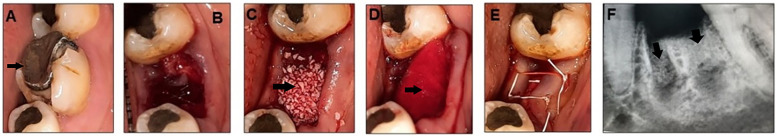
Mandibular left first molar indicated for extraction (black arrow) **(A)**, extraction socket **(B)** socket augmented with Bio-Oss graft materials (black arrow) **(C)** and covered with a conform collagen membrane (black arrow) **(D)** sutured with 4-0 PTFE **(E)** and post-operative periapical radiograph showing the grafted socket (black arrow) **(F)**.

The buccolingual width of the socket was recorded clinically using a periodontal probe and a customized stent fabricated from pre-operative impressions. Based on the surgical notes, graft materials were selected according to the clinic's standard protocol: sockets were filled with either NuOss (ACE Surgical Supply Co., Inc., Brockton, MA, USA) or Bio-Oss (Geistlich Biomaterials, Wolhusen, Switzerland), which were gently condensed from the apical region. The grafted sites were then covered with a collagen membrane (Conform, ACE Surgical Supply Co., Inc.) and secured with tension-free primary closure using 4-0 PTFE sutures (Cytoplast, Osteogenics Biomedical, Lubbock, TX, USA) (Figures 1, 2).

### Post-operative care and follow-up

**Figure 3:**
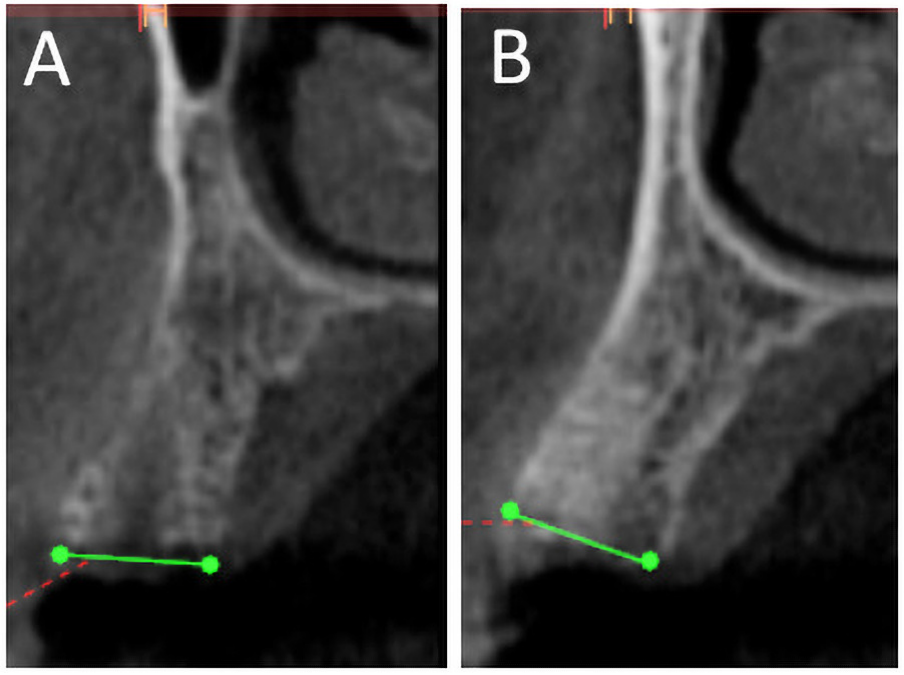
CBCT scan after 6 months for nuOss **(A)** and Bio-Oss **(B)** augmented sockets showing more radiopacity for Bio-Oss sites.

All patients received standardized post-operative care that included: systemic antibiotics (500 mg Amoxicillin TID for 7 days), analgesics (Ibuprofen 600 mg BID), and 0.12% chlorhexidine gluconate rinse (BID for 7 days, initiated 48 hours post-surgery). The sutures were removed after 14 days. Patients were instructed to avoid aggressive brushing near the surgical site for 4 weeks. Patients were recalled after 6 months for CBCT scans using the same machine and settings as the pre-operative scan. To assess changes in buccolingual width and buccal bone thickness, preoperative and 6-month postoperative CBCT scans were superimposed using 3D Slicer version 3.6 (https://www.slicer.org). Measurements of buccolingual ridge width and buccal bone thickness were then performed on matched cross-sections to ensure consistency and accuracy over time. Post-operative CBCT scans were used to assess radiographic density at the grafted sites (Figure 3). Standardized regions of interest (ROIs) were selected at the center of each socket on cross-sectional images using the built-in analysis tools of the CBCT software. Gray scale values within these ROIs were recorded to reflect relative radiopacity of the grafted areas. The same slice orientation and measurement depth were maintained across all scans to ensure consistency. The mean gray values for each group were calculated and included in the results to provide a semi-quantitative assessment of bone density at the grafted sites.

### Re-entry surgery and implant placement

**Figure 4:**
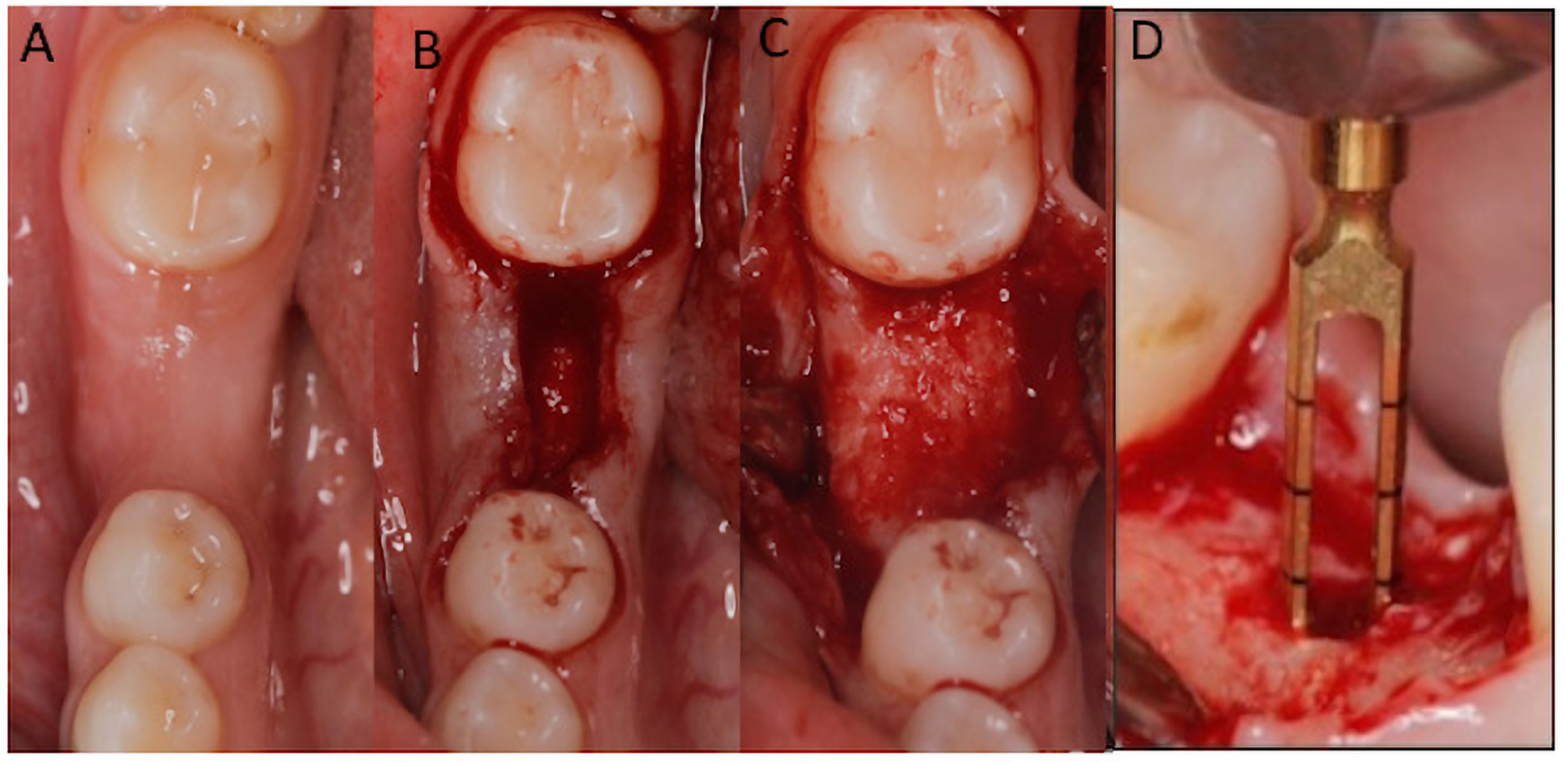
After 6 months **(A)**, full thickness flap was reflected **(B,C)** and 2.0-mm internal diameter trephine bur was used to obtain bone core biopsies **(D)** before implant bed preparation.

After 6 months of healing, a surgical re-entry procedure was performed. Following the application of local anesthesia, crestal and intrasulcular incisions were made and mucoperiosteal flaps were reflected to allow access to the alveolar ridge. A total of 18 biopsies (nine Bio-Oss and nine NuOss) were harvested for histological evaluation from the recruited patients. A trephine bur with a 2.0-mm internal diameter was used to take the core biopsies, which were approximately 8 mm in length. Specimens were marked to identify the coronal and apical ends. The bone cores were coded and fixed in a 10% neutral buffered formalin solution. Osteotomies were completed post-biopsy, and dental implants were placed per manufacturer guidelines and implant planning protocols (Figure 4).

### Histological analysis

Bone cores underwent decalcification using 10% EDITA solution, then dehydration process was conducted by immersion in 90% ethanol then the specimens were embedded in paraffin blocks. Each paraffin block was cut into five sections (3 μm-thick). The sections were stained with hematoxylin and eosin (H & E). Histological and histomorphometry were performed blindly on coded sections under an optical microscope (Nikon Eclipse E600; Nikon, Ltd., Tokyo, Japan) equipped with imaging and analytical software (NIS-Elements; Nikon).

### Histomorphometric analysis

Histological sections were analyzed using calibrated images captured at 20× and 40× magnification with H&E staining. Measurements were performed using ImageJ 1.53c (*N*ational Institutes of Health, USA) (28) image analysis software. A region of interest (ROI) was defined to standardize the analysis, and five random fields of view within this ROI were selected for each specimen. The total tissue area was measured in square micrometers (μm²), and three main components were identified and quantified: new bone formation, residual graft particles, and soft tissue/marrow spaces.

New bone formation was identified by the presence of mineralized tissue that stained pink (eosinophilic) osteocytes within lacunae and revealed characteristic lamellar structure. Residual graft particles were distinguished by their lighter pink staining, characteristic morphology, and empty lacunae. Soft tissue and marrow spaces were identified as the remaining areas containing connective tissue, blood vessels, and cellular components stained purple (basophilic).

The percentage of each component was calculated as:

- Percentage of new bone = (Area of new bone/Total tissue area) × 100.
- Percentage of residual graft = (Area of graft particles/Total tissue area) × 100.
- Percentage of soft tissue = (Area of soft tissue/Total tissue area) × 100.

All measurements were performed by two calibrated examiners, and the mean values were used for statistical analysis. Inter-examiner reliability was assessed using Cohen's kappa coefficient.

### Statistical analysis

Data analysis was performed using IBM SPSS Statistics software (Version 28.0, IBM Corp., Armonk, NY, USA). The normality of the data was assessed using the Shapiro–Wilk test, confirming that the data was normally distributed. The data was expressed as the mean and standard deviation. The *T*-test was used to compare clinical and radiographic measurements before and after augmentation and between the two groups. Statistical significance was set at *p* < 0.05.

## Results

Eighteen patients (6 males and 12 females, aged 23–45 years) were enrolled in the study. The extraction sockets in both groups were carefully matched for size and anatomical sites to ensure comparability of the experimental conditions. This matching approach controlled potential variations that could impact healing and bone regeneration processes.

### Clinical and radiographic observations

All experimental sites healed uneventfully after the tooth extraction and socket augmentation. Four weeks after healing, all augmentation sites showed complete soft tissue healing. In all cases, the grafted sites were able to support the implant placement. After six months, CBCT scans showed more gray scale value in Bio-Oss (mean = 121.61, Min = 93, Max = 144) compared to NuOss groups (mean = 67.223, Min = 43, Max = 90). Inter-examiner reliability was assessed using Cohen's kappa coefficient, which demonstrated a very good agreement (*κ* = 0.9).

**Figure 5:**
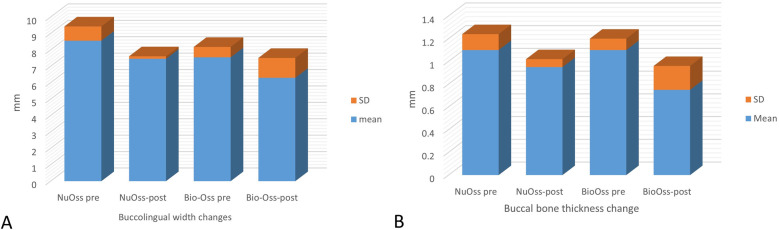
Graphical presentation showing **(A)** changes in buccolingual ridge width in the nuOss and Bio-Oss groups (mean ± SD) and **(B)** changes in buccal bone thickness in the nuOss and Bio-Oss groups (mean ± SD).

The mean change in buccolingual width in the NuOss group revealed a reduction from 8.56 ± 0.87 mm pre-augmentation to 7.46 ± 0.152 mm post-augmentation, which was statistically non-significant (t = 2.1573, *p* = 0.0972). Similarly, the Bio-Oss group demonstrated a reduction from 7.55 ± 0.63 mm pre-augmentation to 6.2 ± 1.2 mm post-augmentation, which was also statistically non-significant (t = 1.3995, *p* = 0.2966) (Figure 5A).

Comparative analysis showed that both groups exhibited comparable mean reductions in buccolingual width, with NuOss group showing a reduction of 1.1 ± 1.04 mm and Bio-Oss group showing a reduction of 1.25 ± 1.06 mm. The inter-group comparison was not statistically significant (t = 0.0213, *p* = 0.9843), indicating similar dimensional changes across both grafting materials.

The mean change in buccal bone thickness in the NuOss group revealed a reduction from 1.1 ± 0.14 mm pre-augmentation to 0.95 ± 0.07 mm post-augmentation, which was statistically non-significant (t = 1.3553, *P* = 0.3081). The mean change in buccal bone thickness in the Bio-Oss group revealed a reduction from 1.1 ± 0.1 mm pre-augmentation to 0.75 ± 0.21 mm post-augmentation, which was statistically non-significant (t = 1.9166, *P* = 0.195) (Figure 5B). Comparative analysis showed that both groups exhibited comparable mean reductions in buccal bone thickness, with NuOss group showing a reduction of 0.15 ± 0.21 mm and Bio-Oss group showing a reduction of 0.3 ± 0.2 mm. The inter-group comparison was not statistically significant (t = 0.6165, *p* = 0.601), indicating similar dimensional changes across both grafting materials.

### Histological findings

**Figure 6:**
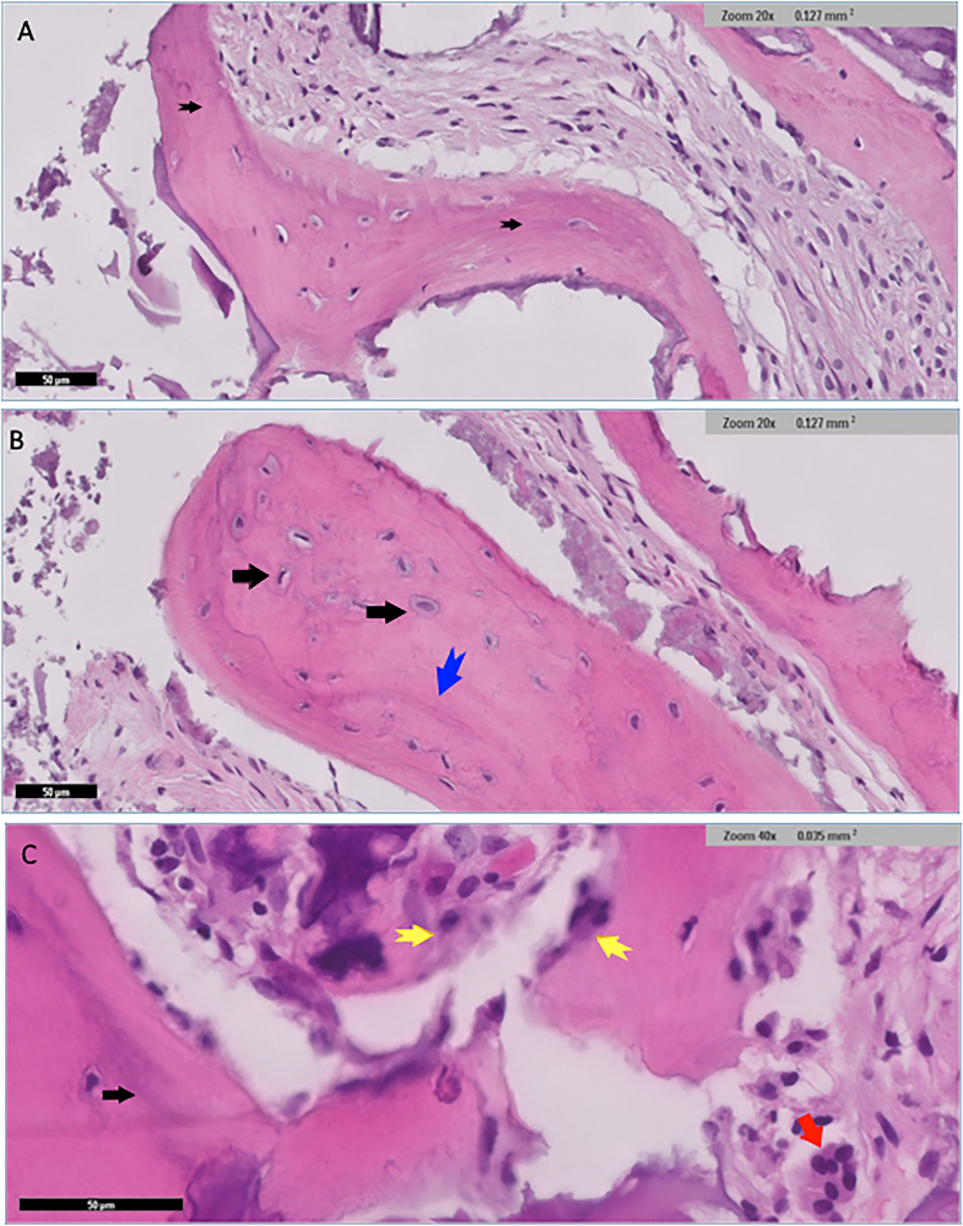
Histological findings for nuOss group showed scattered variably sized fragments of grafted bone embedded in dense fibrocollagenous stroma (black arrows) **(A)** well-preserved osteocytes were identified within the otherwise homogenous pink bone that lacked resting lines (black arrows) **(B)** the bone showed evidence of maturation in places blue arrow in **(B)** and black arrow in **(C)**. Fragments of osteoid rimmed by well-developed osteoblastic rimming (original magnification ×40, yellow arrows) **(C)** Small fragments of residual grafted materials intimately surrounded by osteoclast giant cells (red arrow) were also present **(C)**.

Histological examination for NuOss group revealed a loose collagenous background that supports an admixture of a viable lamellar cortical bone and inorganic matrix. The later shows focal partial integration with the native mature bone. High-power photomicrographs showed partial integration between the native lamellar cortical bone and the added bone graft material. Osteoclasts were identified at the edges of the integrated bony fragments in places (arrow) the rest of the section showed scattered fragments of the added grafted bone. variably sized fragments of grafted bone embedded in dense fibrocollagenous stroma were noted and well-preserved osteocytes were identified within the otherwise homogenous pink bone that lacks resting lines. The bone also displayed evidence of maturation in places (Figure 6).

**Figure 7:**
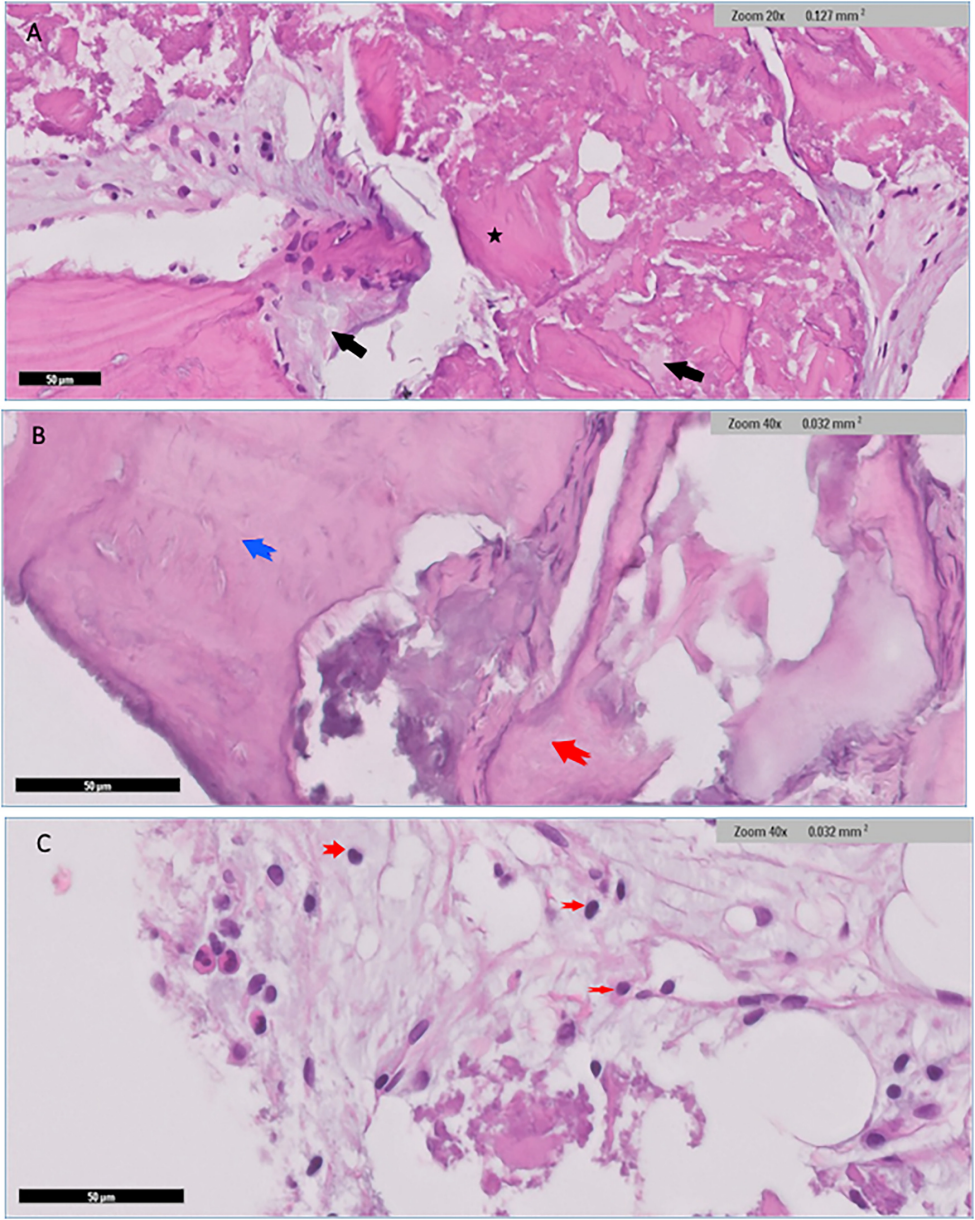
Histological findings for Bio-Oss group revealed admixture of native mature lamellar cortical bone with grafted bone (black arrows), and with evidence of minimal focal integration **(A)** Other areas demonstrated well preserved lamellar cortical/grafted bone integration with evidence of viable osteocytes *(A) Cellular immature/ woven bone was observed (blue arrow), features of grafted bone that were devoid of cells showing evidence of integration (red arrow) **(B)** Clusters of neutrophils (red arrows) were also seen in myxoid background **(C)**.

Low power histological examination of the Bio-Oss group revealed admixture of native mature lamellar cortical bone and grafted bone. There was only focal minimal evidence of integration in some areas, while in others, preserved well-preserved lamellar cortical/grafted bone integration with evidence of viable osteocytes, resting lines and evidence of focal maturation was seen. Clusters of neutrophils were also present in myxoid background. Cellular immature/ woven bone was observed. Hypocellular fibrous tissue supporting fragments of grafted bone that are devoid of cells showing evidence of integration with mature lamellar cortical bone in several places within the section. scattered irregular fragments of grafted bone supporting well-preserved nuclei were also identified in places. The otherwise well-vascularized fibrocollagenous stroma also contains scattered macrophages. Few giant cells were seen at the edges of the section in places (Figure 7).

Histomorphometric analysis of the current groups delineated distinct differences between Bio-Oss and NuOss bone grafts after 6 months of socket preservation. Bio-Oss specimens showed lower new bone formation (37.5 ± 2.5%) compared to NuOss (52.5 ± 2.5%) while maintaining higher amounts of residual graft material (42.5 ± 2.5% vs. 27.5 ± 2.5% respectively). The soft tissue component showed relatively similar values between both materials, with Bio-Oss showing slightly lower percentages (17.5 ± 2.5%) compared to NuOss (22.5 ± 2.5%).

**Figure 8:**
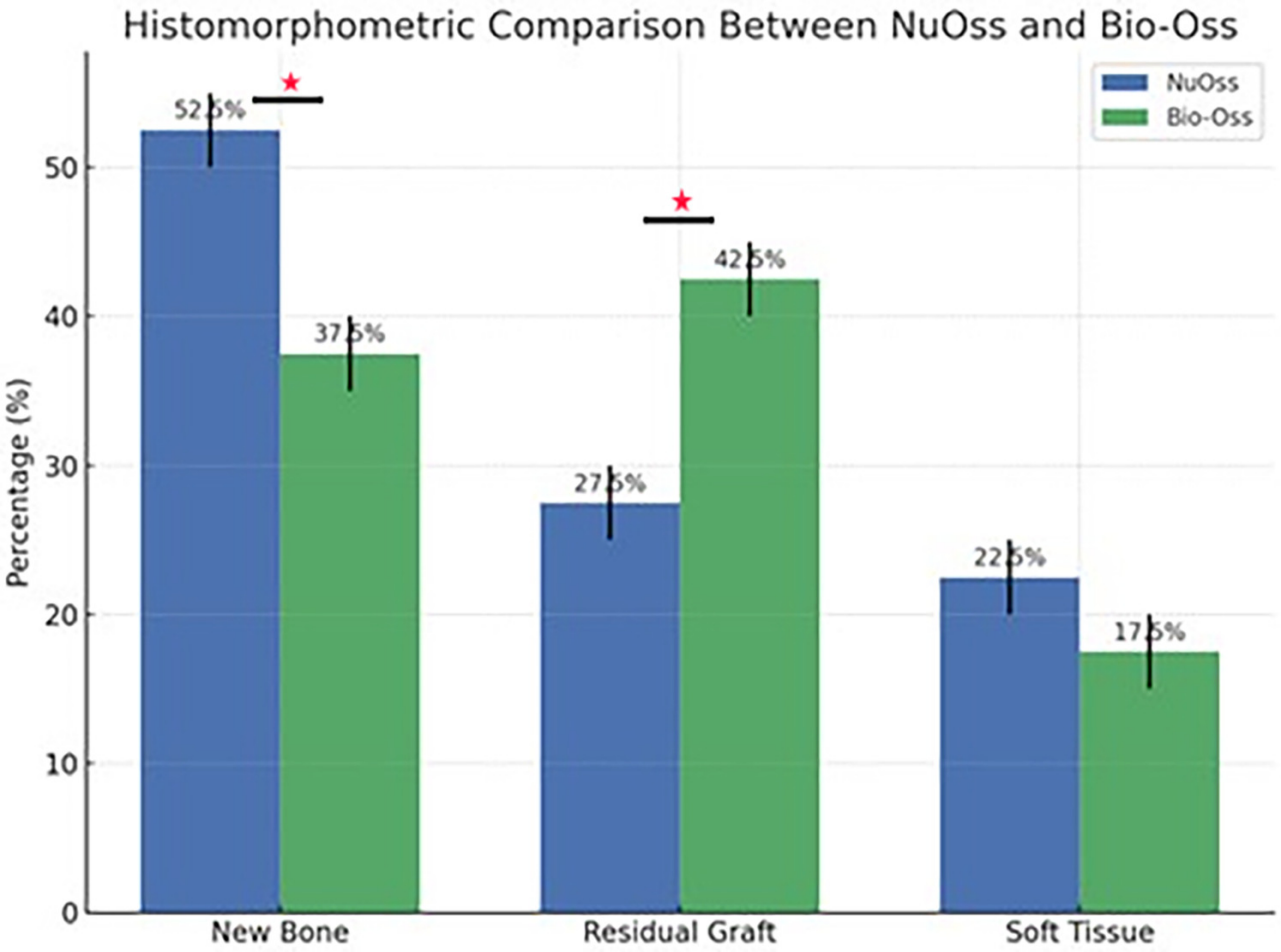
Graphical presentation showing the histomorphometric results between the nuOss and Bio-Oss groups. *statistically significant differences.

Statistical analysis was performed using independent t-tests to compare the histomorphometric measurements between Bio-Oss and NuOss specimens. The comparison of new bone formation revealed significantly higher values in NuOss (52.5 ± 2.5%) compared to Bio-Oss (37.5 ± 2.5%) (*p* = 0.0021, 95% CI: 10.2–19.8%). Analysis of residual graft material showed significantly lower amounts in NuOss specimens (27.5 ± 2.5%) compared to Bio-Oss (42.5 ± 2.5%) (*p* = 0.0018, 95% CI: 10.5–19.5%). The difference in soft tissue proportions between NuOss (22.5 ± 2.5%) and Bio-Oss (17.5 ± 2.5%) did not reach statistical significance (*p* = 0.0892, 95% CI: −1.2–11.2%) (Figure 8).

These findings suggest that NuOss is characterized by a significantly higher bone formation potential and more rapid resorption compared to Bio-Oss at 6 months post-grafting, while maintaining comparable soft tissue proportions.

## Discussion

Evaluation of NuOss and Bio-Oss as bone graft materials has shown varying results across studies. Our CBCT findings after six months demonstrated higher radio-opacity in Bio-Oss compared to NuOss groups, though both materials showed comparable mean reductions in buccolingual width and labial bone thickness.

Histologically, our study revealed different patterns of tissue response between the materials. In the NuOss group, we observed loose collagenous background with viable lamellar cortical bone and partial integration with the native mature bone. Osteoclasts were present at the edge of integrated bony fragments, consistent with normal bone remodeling processes.

In the Bio-Oss group, we found minimal focal integration between native mature lamellar cortical bone and grafted bone, with evidence of viable osteocytes and resting lines. This observation aligns with Artzi et al. (29), who reported cellular presence in extraction sites filled with Bio-Oss. In agreement with previous studies (30, 31) Our findings of clusters of neutrophils and scattered macrophages, with few giant cells at section edges, parallel the inflammatory responses. However, the inflammatory response patterns vary in the literature. While Piatteli et al. (32), Molly et al. (33) and Degidi et al. (34) reported no significant inflammatory cell presence with Bio-Oss, our observations showed a moderate inflammatory response. This variation might be attributed to methodological differences and surgical techniques, as suggested by previous studies.

Regarding bone formation and resorption, our histological findings showed evidence of maturation in both groups, though with different patterns. This observation contributes to the ongoing discussion about Bio-Oss resorption rates, where some studies report rapid replacement with host bone (35), while others suggest a slower resorption process (36–38). The presence of osteoclasts in our samples aligns with Piatelli et al. (39) observations regarding Bio-Oss resorption capacity.

The absence of significant foreign body reaction in the NuOss group suggests good biocompatibility, contrasting with the occasional giant cell presence observed in the Bio-Oss group. This finding adds to the complex picture presented by Commack et al. (40) regarding foreign body reactions to Bio-Oss. This findings align with previous research on socket preservation techniques. While both Bio-Oss and NuOss demonstrated the ability to reduce post-extraction dimensional changes, complete preservation of original ridge dimensions was not achieved, as evidenced by the observed mean reductions in buccolingual width and labial bone thickness. This outcome corresponds with findings reported by Fickl et al. (41) who demonstrated that while deproteinized bovine bone mineral (DBBM) could limit post-operative tissue shrinkage, it could not completely prevent tissue alterations following tooth extraction. Similarly, Nevins et al. (25) reported that DBBM-filled extraction sockets showed better outcomes compared to untreated sockets but still could not maintain the baseline height of the bone crest entirely.

While guided bone regeneration (GBR) techniques have shown improved ridge dimensions when compared to extraction alone (42, 43), complete ridge preservation remains challenging. Simon et al. (44) found that original contours were best maintained when additional GBR was performed on the buccal and coronal portions of the alveolus, suggesting that supplementary overlay grafting might be necessary for optimal aesthetic outcomes.

Studies have shown that bovine bone mineral with 10% purified porcine collagen reduces alveolar bone resorption and promotes new bone formation in canine extraction sockets (45, 46). However, histomorphometric evidence of early healing patterns in human extraction sites using this material is limited to only two studies (47, 48). Alkan et al. (49) compared the efficacy of enamel matrix derivatives (EMD) and Bio-Oss Collagen in human extraction sockets. While EMD sites showed higher implant stability quotient (ISQ) values at one and three months, both materials demonstrated comparable bone formation patterns. In contrast to our findings, Alkan et al. (49) reported no inflammatory cell infiltration was observed histologically, both groups showed no, suggesting that both EMD and Bio-Oss Collagen are effective for socket preservation before implant placement.

Studies examining socket preservation techniques have shown varying results regarding the timing of implant placement and regenerated bone quality. Heberer et al. (47) found higher rates of new bone formation in ungrafted vs. bovine bone mineral-grafted sockets after 12 weeks, suggesting delayed bone formation in grafted sites. Alkan et al. (49) observed new bone formation surrounding Bio-Oss Collagen particles, consistent with several previous studies (22, 50). Other materials such as demineralized dentin graft, platelet-rich fibrin, freeze-dried bone allograft, corticocancellous porcine, and autogenous bone have shown promising results, with some achieving over 50% new bone formation in clinical studies (51). The use of platelet-rich fibrin (PRF) combined with other graft materials, such as autologous dentin grafts, has been shown to improve radiographic bone fill and socket measurements, suggesting enhanced regenerative outcomes (52). Advanced techniques like the use of injectable bone repair materials and hydrogel systems are being explored for their ability to accelerate bone healing and improve patient-reported outcomes (53). Demineralized tooth dentin has been used for socket preservation and demonstrated higher bone regeneration compared to traditional methods (54).

The efficacy of deproteinized bovine bone for implant osseointegration remains controversial. While some studies support its use, others have found limited contribution to vital bone-implant contact (55, 56). A previous study indicated that implants placed in augmented sites showed survival rates comparable to those in native bone (57). Previous studies (58, 59) reported no significant differences in implant survival between guided bone regeneration and control groups.

Implant stability is achieved through primary and secondary stability. Primary stability is mechanical and depends on the quality of the bone and the implant design, while secondary stability is biological and results from osseointegration (60, 61). The use of graft materials like NuOss, that promotes new bone formation while minimizing residual graft material, can potentially enhance secondary implant stability, leading to improved clinical outcomes. This is particularly important in cases where immediate or early loading of implants is desired, as it reduces the healing period and enhances the predictability of implant success (62, 63).

Reducing residual graft material is beneficial as it minimizes the potential for foreign body reactions and allows for more natural bone remodeling. This can lead to a more stable bone-implant interface, which is essential for long-term implant success (62). Moreover, the reduction in residual graft materials that were observed in NuOss group can facilitate better bone integration with the implant (64).

While decalcified H&E staining was used in this study for its accessibility and generalizability, further studies using ground sections may reduce the presence of artifacts such as voids and enhance structural clarity.

To our knowledge, there is limited literature that directly compares NuOss to other graft materials in socket preservation. This study presents a novel, integrated approach by simultaneously evaluating clinical, radiographic, and histological parameters in comparing the outcomes of two xenografts Bio-Oss and NuOss in socket preservation. While many previous studies have focused solely on either dimensional changes or histological findings, our methodology allows for a more comprehensive assessment of bone healing over a 6-month healing period.

This study has several limitations. First, the sample size was relatively small, with only eighteen patients, which may limit the generalizability of the findings. Future studies with larger samples are highly recommended. Second, the study employed a non-randomized design, which may introduce selection bias and limit the robustness of the results. Without randomization, there is a potential risk of selection bias, as group assignment may be influenced by clinical factors or practitioner preference. This can affect the internal validity of the study and may confound the outcomes, especially if baseline characteristics differ between groups. Although efforts were made to match cases based on socket characteristics and anatomical location, residual confounding cannot be ruled out. Future randomized controlled trials are recommended to eliminate allocation bias and strengthen the causal conclusions drawn from treatment comparisons. Third, the use of only two specific bovine bone minerals (Bio-Oss and NuOss) restricts the applicability of the results to other grafting materials.

## Conclusion

This study demonstrated that both Bio-Oss and NuOss are effective in maintaining alveolar ridge dimensions following tooth extraction, with NuOss showing superior outcomes in terms of new bone formation, lower residual graft material, and reduced inflammatory response. These findings suggest that NuOss may be a more favorable option for socket preservation in clinical practice. Further studies with larger sample sizes and extended follow-up periods are recommended to confirm these results and evaluate the long-term stability of the regenerated bone.

## Data Availability

The original contributions presented in the study are included in the article/Supplementary Material, further inquiries can be directed to the corresponding author.
